# Supplementary material for: Metabolome Shift in Both Metastatic Breast Cancer Cells and Astrocytes Which May Contribute to the Tumor Microenvironment
Source: Int J Mol Sci. 2021 Jul 11;22(14):7430. doi: 10.3390/ijms22147430 (PMC8308096; doi:10.3390/ijms22147430)
Supplement: Supplementary file 1 [file ijms-22-07430-s001.zip › supplmentary FigS1.pdf]

Figure S1

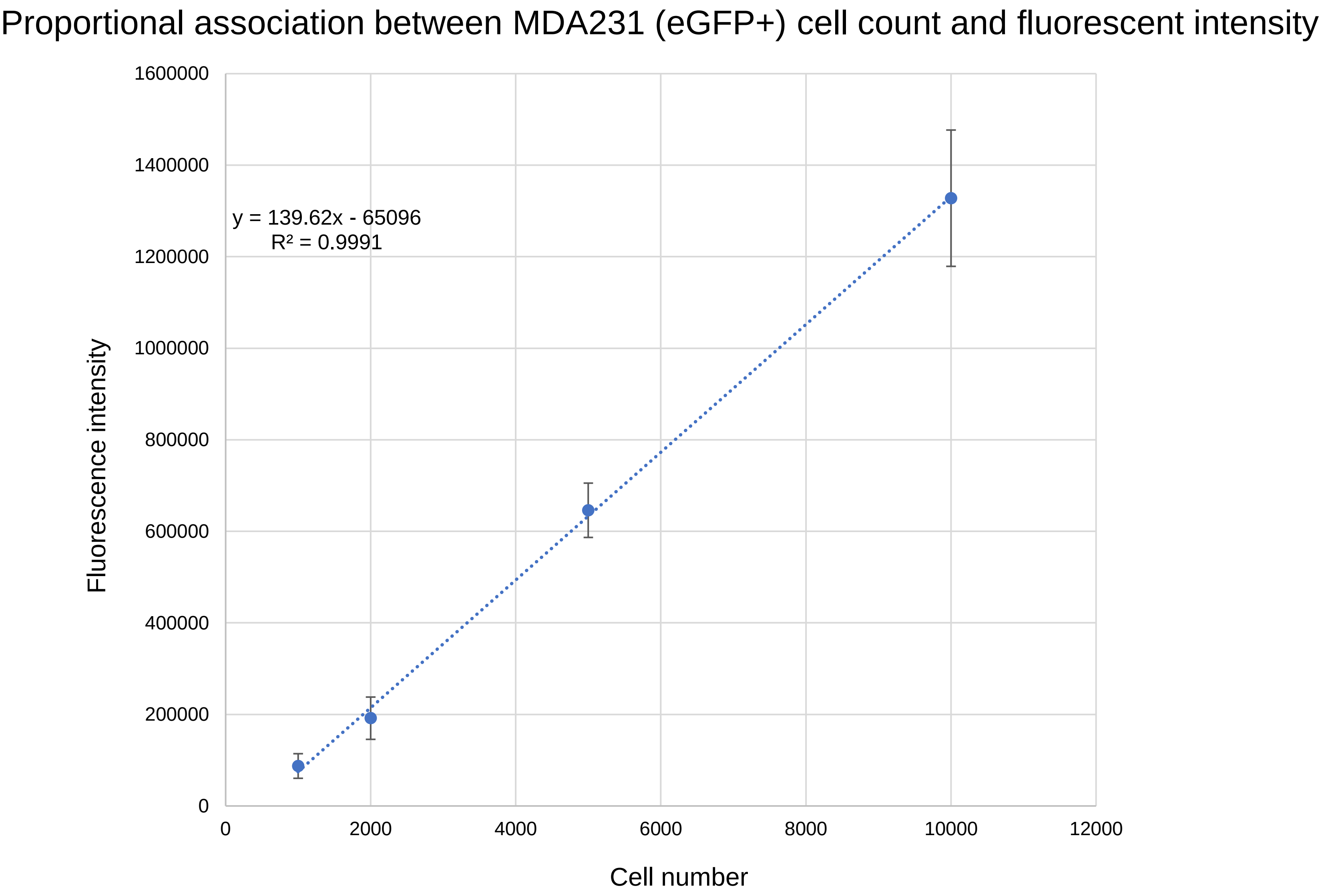

MDA231 (eGFP+) was suspended in Phenol Red Free medium and seeded onto 96 well plates. Four cell numbers were prepared at 1000, 2000, 5000 and 10,000 cells. After 24h of seeding, the medium was removed and replaced with PBS, and the eGFP fluorescence intensity was measured. Fluorescence intensity is expressed as mean ± SD (n=6).
